# Possible Role for Imagery-Based Therapy in Managing PTSD in Pakistani Women Experiencing Domestic Abuse: A Pilot Study Using Eidetic Therapy

**DOI:** 10.3390/ijerph18052478

**Published:** 2021-03-03

**Authors:** Mehwish Kamran Ehsan, David L. Rowland

**Affiliations:** 1 Department of Professional Psychology, Bahria University, Islamabad 44000, Pakistan; neha-1@live.com; 2 Department of Psychology, Valparaiso University, Valparaiso, IN 46383, USA

**Keywords:** domestic abuse, women, PTSD symptoms, intervention, therapy, cultural factors, eidetic therapy, imagery-based therapy, sexual abuse, psychological abuse, physical abuse

## Abstract

Domestic abuse of women is a serious problem worldwide that has economic, physical, and psychological consequences, yet in many countries and cultures, victims often have little access to psychological support. Using a pre-post design, we investigated the effects of psychological intervention using an imagery-based therapy in women showing post-traumatic stress disorder (PTSD) resulting from spousal domestic abuse. Forty women, referred from outpatient clinics in Pakistan and meeting inclusion criteria, underwent individual trauma counseling for 10–12 weeks using the principles of Eidetic Therapy, an imagery-based therapy that circumvents heavy reliance on verbal skills and narratives. Women showed significant reductions in PTSD by the end of treatment. Predictors of treatment gains included type of abuse, PTSD level at the outset of therapy, and years in the relationship. Neither economic resources or literacy, nor abuser or victim characteristics, predicted the amount of improvement. In conclusion, therapy was associated with a reduction in PTSD symptoms regardless of literacy level of participants. This reduction in PTSD was notable because, unlike many situations involving spousal abuse, these women were generally not in a position to leave their relationship, and hence the women might have experienced continued exposure to abuse. Context/cultural-based explanations for these findings are presented and discussed.

## 1. Introduction

### 1.1. Domestic Abuse and Its Prevalence

Although an age-old problem, domestic abuse (DA)—verbal, emotional, physical, economic, and sexual—has been increasingly recognized as one of the most serious challenges facing women today [1,2]. Specifically, the Intimate Partner Violence (IPV) survey (World Health Organization: WHO [3]) based on 24,000 women from 10 countries has confirmed the widespread prevalence of DA worldwide: Women experiencing physical violence ranged from 13–61%, severe physical violence by a partner from 4–49%, sexual violence from 6–59%, and emotional abuse from 20–75%. Other global research has revealed comparably high percentages, sometimes as high as 60% (e.g., [4]). Although violence against women is considered a violation of human rights, WHO [5] estimates that over one billion women lack legal protection against violence by an intimate partner or family member.

#### Domestic Abuse in Pakistan

Although prevalence data from probability samples are scant, studies suggest a fairly high rate of DA in Pakistan. The popular press intimates that Pakistan falls on the higher side of comparison countries [6,7]. In addition, data-based studies report wide ranges of DA prevalence in Pakistan, for example, 21–50% of women for any type of DA [1], and 50–70% for DA (including physical) from either the intimate partner or another person over their lifetime [8,9,10]. Even these rates might underestimate the true prevalence of DA, given its covert nature and probable underreporting. Pakistan’s problem, however, is not unique to this global region but reflects a larger problem typical of the socio-cultural milieu of central/south Asia [11].

DA in Pakistan cannot be attributed to any single cause. Rather, stressful financial conditions, perception of the wife’s infertility, cultural practices, illiteracy, and conflict with husband, family, and in-laws are contributing factors [12]. Furthermore, a strong patriarchal system that affords lower status to women means that legal protections for female DA victims in Pakistan are often weak, unenforced, or non-existent [8,13].

### 1.2. Consequences of Domestic Violence

DA is harmful in a variety of ways, affecting women’s physical, psychological, and economic well-being [14,15]. Specifically, DA may have deleterious effects on women’s physical health through several pathways, including actual physical injury, increased reproductive problems, and greater risk for chronic diseases [16,17]. Furthermore, women’s responses to such violence may include psychological distress/dysfunction (e.g., anxiety), changes in cognitive schemata (e.g., loss of assumption of invulnerability; negative beliefs about self); and relational disturbances (e.g., difficulty with trust and attachment) [18]. For example, 75% of female DA victims show severe anxiety/depression, and many exhibit diminished self-esteem, difficulty with emotional self-regulation, and social isolation [19,20]. Other effects may include self-harm, addiction, poor parenting, and issues regarding intimacy, often exacerbated when the woman continues to live in a stressful, trauma-producing environment [21,22,23].

#### Posttraumatic Stress Disorder (PTSD) as a Consequence of Domestic Abuse

The effects of DA often appear as posttraumatic stress disorder (PTSD) [21,24]. PTSD typically entails the perception of physical or severe psychological threat or injury, including death, as an individual faces a traumatic event [25]. The consequence of such experiences may include traumatic stress, intrusion or re-experiencing, negative alterations in mood or cognitions, and increased arousal symptoms. As a general stress response, acute reactions may manifest in the form of disturbing memories, avoidance behaviors, physical symptoms, general loss of interest, and sleep difficulties [26]. Indeed, there is evidence to suggest a neurobiological basis for PTSD via over-activation of the cortisol stress response system involving the hypothalamic-pituitary-adrenal axis. Specific PTSD symptomology such as hyperarousal, trauma nightmares, and sleep disturbances, has been associated with high noradrenergic activity, probably through the stimulation of α -1 adrenergic receptors in the brain prefrontal cortex [27].

Consistent with these effects, women who experience DA often suffer from PTSD symptoms (or complex trauma when the experiences are ongoing), feeling distrust, abandoned, guilty, and betrayed, and showing signs of hypervigilance, avoidance of traumatic triggers, flashbacks of events, anxiety, nightmares, imagery, numbness, and insomnia [28]. As a prime example, Yoshihama and Horrocks [20] have demonstrated that women who faced ongoing emotional DA reported higher levels of re-experiencing and avoidance symptoms typical of PTSD than non-victims.

### 1.3. Interventions for PTSD

Various approaches have been implemented to address DA against women in Pakistan, including interventions at the community, family, and individual levels [29]. Community and family interventions, which strive to change attitudes and behaviors, may target community stakeholders, extended families, and spouses/men in order to reduce the overall prevalence of DA [30,31]. Such approaches are critical to developing enduring strategies by increasing the valuation of women by altering deeply embedded socio-cultural traditions and practices. Such programs may also lead to further legislative protections for women and eventual reform. Nevertheless, when legalities clash with culture and religion, abuse-event reporting and enforcement are often weak or ineffective [32,33]. Furthermore, it is naïve to assume that community and family interventions will achieve rapid transitions in attitudes and practices in Pakistan; more likely, behavioral changes will occur over generations, not months or years.

In the meantime, women continue to suffer with PTSD from DA, and even when/if community programs begin to show results, DA will persist in situations/areas where traditional norms are deeply engrained. Thus, more immediate individual-level interventions may be warranted to help women manage symptomatology that affects their psychological health and severely disrupts their daily functioning [34]. In general, treatments for PTSD have been both pharmacological and psychological. For example, antidepressants may be effective in managing PTSD, although this approach is primarily a symptomatic one (e.g., [35]). Several psychologically based trauma interventions have also shown promise [36]. For example, trauma-based cognitive behavioral therapy (CBT) and eye movement desensitization and reprocessing have garnered considerable support using randomized trials for PTSD reduction [37]. Furthermore, given the imagery-based nature of PTSD flashbacks, one important element that may enhance the effectiveness of therapy is the use of mental imagery as a therapeutic tool, particularly insofar as it might complement or eclipse the verbal-based elements in PTSD treatment [38,39,40].

#### Eidetic Psychotherapy (EP) as an Image-Based Approach to PTSD Treatment

One type of therapy that has been used successfully in the treatment of a variety of psychological disorders, including phobias, drug addiction, eating disorders, pain, anxiety, sexual issues, and depression, is Eidetic Psychotherapy (EP) [41,42,43,44]. EP has also been used successfully in the treatment of PTSD as demonstrated through 30 case histories reported by Ahsen [45]. EP is an experiential-oriented therapy that relies on elicitation of mental imagery—characterized as “eidetic”—that enables self-examination so the individual can come to understand why she behaves/feels/responds in certain ways [46,47]. The potential advantages of imagery over verbal processing for PTSD include (1) that PTSD itself often evokes strong imagery-based recall of events [48]; (2) imagery is less likely to be influenced by education/literacy than therapies requiring language proficiency, particularly when therapy is carried out in the person’s 2nd or 3rd language; and (3) imagery largely circumvents the cultural biases embedded in linguistic expression [46], see also [49] and therefore may represent a more culturally friendly approach for treatment in non-Western populations. In EP—itself having roots in South/Central Asian culture—the resolution of the patient’s issues emphasizes examination of the images associated with the traumatic events through eidetic “seeing” as opposed to thinking, representing a counterpart to cognitive therapy which places strong emphasis on verbal self-narratives. Specifically, as the image is formed, the client—under the guidance of the therapist—interacts with it, allowing it to unfold and experiencing its emotional meaning. Such interactions can lead to self-discovery, and manipulation of the image can affect both emotional and bodily responses, as well as the meanings associated with them. The use of imagery-based therapy, as is done in EP, has long been acknowledged as having a role for psychological disorders [44,50], including in the resolution of anxiety, depression, PTSD, phobias, obsessive-compulsive disorder, and eating disorders [25].

EP has been proven an effective short-term approach offering several advantages in clinical settings: as an experienced-based approach aimed at resolving long-term negative emotional patterns, it strongly supports patients’ self-reliance and insight, enabling them to recognize somatic and emotional reactions as well as gaps in consciousness [51]. At the same time, the procedure for the therapist is simple and direct, can be extended to patients with a variety of psychological disorders, and is based on the idea that treating trauma through imagery can help establish the connection between the causal event and symptoms which, once achieved, can help to re-interpret the problem and mitigate the symptoms [41,44].

### 1.4. Rationale and Aims

Although various psychotherapies have been used effectively to help individuals suffering from PTSD, such techniques have been applied only sparingly in populations where mental healthcare has low priority and where religious and cultural values differ greatly from those of the West, the cultural framework in which most therapies have developed. Although traditional psychotherapies such as CBT have demonstrated effectiveness in Pakistani populations for depression [52], there is a dearth of indexed literature on the non-pharmacological treatment of PTSD in Pakistani women, particularly when it results from DA. Furthermore, a recent systems analysis of psychotherapies in Pakistan indicated the need to “make numerous adjustments in CBT” (thus termed Culturally Adjusted CBT) in order to make it acceptable and workable within these communities [53], including the use of religious practices as part of the therapy [54]. In other parts of the world, including the USA, issues surrounding literacy and regional traditions have prompted cultural adaptations in therapy for the treatment of chronic pain and depression [55,56,57].

We further note that traditional assumptions surrounding illness (especially psychologically based problems) and causality in Western systems are not necessarily applicable to other cultures. In fact, the argument has been increasingly made that psychological findings/therapies based on Western studies may have little generalizability to much of the rest of the world [58,59] and that to assume so is likely to lead to erroneous conclusions. Specifically, culture is known to play a critical role in men’s and women’s expectations and experiences regarding health, illness, and, in particular, psychological problems, including the way individuals describe, interpret, and ascribe meaning to the problem [60,61,62,63]. Indeed, the interpretation/meaning-making process of a problem within a cultural tradition is often as important for the individual as the behavioral/physical signs and symptoms of the disorder itself. For example, common interpretations in non-Western cultures of negative health and well-being outcomes are often related to judgments about oneself (e.g., bad things happen to bad people so I must be bad) and the prevailing morality (e.g., it is proper to submit to my husband and wrong to contradict him).

Given the above framework, the continuing development and improvement of methods for treating PTSD that can be readily adapted to various populations and cultures—particularly where literacy, verbal skills, and cultural beliefs may represent significant obstacles—is strongly warranted. Furthermore, given that EP was originally developed as a more culturally friendly approach, its usefulness in the treatment of psychological issues—thus far only minimally tested—in non-Western contexts is worth assessing, particularly in regions of the world such as Pakistan where, due to precarious geopolitical and quality of life conditions, physical and mental trauma represent heavy burdens on the population. Data regarding the effectiveness of EP specifically for the treatment of PTSD could contribute to the existing cross-cultural psychotherapeutic tools for improving women’s psychological health and adjustment. Moreover, the abbreviated format of EP, often consisting of only 5–10 sessions, has the potential to save both time and money, making it an attractive option in environments where personal and public resources for mental healthcare are severely limited.

This study was designed to (1) document PTSD symptoms in women experiencing DA in Pakistan; (2) test the effectiveness of the imagery-based ET in alleviating PTSD symptoms from DA; (3) determine whether a therapeutic process that relies primarily on imagery might be equally effective in women of contrasting literacy skills/levels, a common differentiator of rural and less educated populations in many emerging nations; (4) identify demographic and abuse-related variables associated with greater improvement in response to the therapy.

## 2. Materials and Methods

### 2.1. Participants

Sixty women identified through the outpatient clinics of hospitals (medical/psychiatric/psychological units) located in two major cities in Pakistan, Rawalpindi and Islamabad, were invited through purposive consecutive sampling to participate in a study investigating the remediation effects of therapy on PTSD due to DA. Criteria for participation included: outpatient status; married and living with their husband; between 18 and 64 years, based on ages of greater DA vulnerability [64]; meeting PTSD criteria; not taking medication or currently under psychotherapy treatment for the PTSD; free of major psychosomatic and psychiatric disorders based on assessment using the General Health Questionnaire (GHQ); free of incapacitating chronic physical illness, mental disabilities, or other anomalies that might interfere with therapy (as determined by medical records, consultation with primary healthcare providers, and the semi-structured interview); referral/approval from the patient’s attending psychiatrist or psychologist. Of the 60 candidates, 15 women did not qualify for participation, with another 5 not scoring sufficiently high on PTSD symptomatology. The final sample included 40 women (mean age = 34.3 y; SE = 1.18). To ensure demographic diversity, participants were recruited from both a public/government-funded hospital in Rawalpindi, which serves rural and poorer urban populations (*n* = 20), and a private hospital in Islamabad (*n* = 20) drawing mainly urban and middle/higher socioeconomic patients.

### 2.2. Assessment Instruments

General Health Questionnaire-28. This widely used instrument (Urdu version) screened out participants having major psychiatric and/or psychosomatic disorders, or levels of PTSD that might require pharmacological or psychiatric intervention (https://eprovide.mapi-trust.org/instruments/general-health-questionnaire (accessed on 2 March 2021).

Demographic Information Form. This form collected information about the participant’s age, education, occupation, years of marriage, number of children, husband’s job and education, family income, family structure, and DA types.

Standard Intake History Form. Using a semi-structured interview format, information was collected about the presenting complaints, prior treatments, family environment, and medical, work, and school histories of participants.

Karachi Domestic Violence Screening Scale (KDVSS). The KDVSS, Urdu version [65], was used to verify victimization of DA. The instrument consists of 35 items organized into five subscales: physical abuse; psychological abuse; sexual abuse; abuser characteristics; victim characteristics, with higher scores representing higher levels on the designated subscale. The KDVSS has good internal consistency (0.925) and strong test re-test reliability over one month’s time (0.890). Convergent validity (r = 0.899) and discriminant validity (r = −0.927) support the KDVSS as a discriminating tool for measuring the presence and severity of DA committed by husbands against their wives [65].

PTSD Checklist-Civilian Version (PCL-C). The PCL-C [66] consists of 17 items in which participants indicate how much they have been bothered by a specific symptom over the past month on a 5-point scale (1 = not at all; 5 = extremely) regarding various stressful life experiences. Overall scores range from 17 to 85. Four symptom categories are included: Re-experiencing (items 1–5); Avoidance (items 6 and 7); Numbing (items 8–12); Hyperarousal (items 13–17), but only a single overall index is generated.

PCL-C use in clinical and non-clinical populations has shown high internal consistency, test-retest reliability, and divergent and convergent validity, with optimal cutoff scores of 44 and 50 suggested for diagnosing PTSD among trauma survivors [67,68]. The present study used the Urdu version [69] which included modifications to focus more on specific events as they related to DA. Consistent with previous research, a cut-off of 50 was used.

### 2.3. Procedure and Design

The study received approval from the Ethics Committee of Bahria University, Islamabad, Pakistan. Procedural details, benefits, and risks were explained to participants, including the opportunity for PTSD-related therapy at no charge, with participants providing written informed consent prior to the study. As is customary in Pakistani culture, women were permitted to bring along a friend or supportive family member, but all assessment procedures, including interviews, were administered individually. A sole therapist (one of the co-authors) certified in EP carried out the therapy. This therapist was professionally qualified through academic and practical training (in doctoral training at the time and possessing a master’s degree in clinical psychology), with her services supervised by a certified trainer in ET as well as by qualified faculty in her academic training program. The therapist also ensured adherence to the stepwise progression of treatment noted in Tables S1 and S2 in Supplementary Materials**.**

The study used a pre-post design to determine a role for EP on DA-related PTSD. The study was thus conducted in three phases: Pre-intervention, Intervention, and Post-Intervention, carried out 3 months after therapy ended.

Pre-Intervention. Women provided demographic information, underwent a semi-structured intake interview, were screened for psychiatric disorders using the GHQ, and provided information regarding their DA and PTSD symptoms using the KDVSS and PCL-C, respectively. Eligible participants were then approved for further participation.

Intervention. This phase consisted of 40 min sessions spread over 12–15 weeks, with one session usually scheduled for each week (10–12 sessions total). Details of the treatment plan are provided in Section 2.4 and of the progression through therapy are provided in supplementary documents (Tables S1 and S2 in Supplementary Materials). At the beginning of every session, participants were verbally (and briefly) assessed for PTSD symptoms consistent with the PCL statements. When PTSD symptoms fell in the 0–1 range on most items for the participant, treatment was terminated, accounting for the variable number of treatment sessions across participants. As such, all participants completed the treatment.

Post Intervention. Three months after the intervention ended, participants again completed the PCL-C to assess any reductions in PTSD symptoms and to ensure that effects were not merely fleeting or transient. At this time, the therapist also verified that no intervening treatments were utilized.

### 2.4. Treatment Plan and Progression

A comprehensive “Treatment Manual” based on Ahsen’s Trauma Model [42,43] provided the framework for therapy sessions and progression, including steps involving exploration of emotional and physiological responses to trauma, as follows.

Preliminary Information on Trauma. Information regarding the trauma was gathered, using both verbal and imagery cues relevant to the events. These images were then processed through a series of explorations as noted below.

Conscious Remembrance. During the initial phase of treatment, patients were asked to recall and describe verbally, in a holistic manner, their abuse and the trauma surrounding it in order to provide a starting point for the therapeutic process. This memory was typically limited, lacking in details and sometimes inaccurate.

What Other People Described. In this stage, information was gathered from another eyewitness to the event. However, due to the sensitivity of cases of DA, in some instances, other eyewitnesses were absent, while in others, the person accompanying the client could provide information which, even though not always consistent with the client’s narrative, provided information (cues) considered for discussion within the framework of the treatment.

Recurrent Images of the Patient. In this step, participants were asked to report recurrent images, fixed or in flux, that came to mind while thinking of the traumatic experience related to DA. Participants described and discussed these images, which provided additional information and cues about the traumatic experiences which were important for dealing with the trauma.

Levels of Inquiry. Throughout the treatment process, seven levels of inquiry were used to precisely define the image(s) relating to the traumatic symptoms. In addition, participants identified events that were often precursors to the DA that then became associated with the traumatic experience occurrence. These events and the traumatic experiences themselves were then analyzed through the following levels of inquiry: (1) to recall trauma on a conscious level; (2) during mid-phase, to visualize images related to the impact of traumatic experience; (3) to visualize images, moment-by-moment, which preceded the impact of trauma; (4) as the sequence of images began to unfold, to visualize the images pertaining to the key event which resulted in the trauma; (5) to visualize the images at the start of the day or hours prior to the traumatic experience; (6) to visualize the images that followed the trauma; (7) to visualize the images related to recovery and restoration from trauma.

Application of Accident/Event Trauma Imagery. In order to treat the PTSD, “accident/event trauma imagery” was applied in the intervention phase. Briefly, after identifying physiological and emotional response to the trauma imagery, Steps 5–17 of Ahsen’s model (Supplement S2) were applied [41,42,45]. During this period, the client was able to develop a clearer understanding of the relationship between the abusive events and her (emotional and physiological) responses. This realization, along with the imagery, enabled the process of recovering the symptoms and releasing the negative feelings, thereby providing relief from the strong emotional response. Additionally, by repeating the visual image (carrying the traumatic experience) with verbal narrative, both the physiological and emotional responses to the image began to wane, thus resulting in reduction in trauma symptoms. As symptoms decreased, therapy ended, and the post-intervention session was scheduled.

### 2.5. Statistical Analyses

Changes in PTSD symptoms (as measured by the PCL-C) following intervention were analyzed using repeated measures ANCOVA, with age included as a covariate. Predictors of the amount of reduction in PTSD symptoms as measured by the PCL-C change score were assessed through a series of regression analyses that combined sets of cohesive covariates in separate runs, necessitated by the limited sample size of 40. PCL-C change score was calculated as the pretest PCL score minus the posttest PCL score.

## 3. Results

### 3.1. Description of the Sample and Correlations among Study Variables (Aim 1)

Table 1 provides basic information about the sample, showing characteristics of the participants, including their diversity in age, education, years of marriage, and socioeconomic class. In anticipation of regression analysis, Table 2 shows correlations between predictor covariates and the regression outcome variable (change in PCL symptoms) resulting from intervention. Correlations among covariates were also used to identify non-collinear covariates for inclusion in the regression analysis.

### 3.2. PTSD Symptoms in Victims of Domestic Abuse Following Therapy (Aim 2)

PTSD symptoms were significantly lower at the end of therapy (F [1,38] = 28.4; *p* < 0.001, effect size = 0.43), with symptoms decreasing from pretest levels of 60.9 (SE = 1.37) to post-test levels of 19.8 (SE = 0.23). Of the 40 women, all showed at least some benefit from intervention, with the average change score of 41.1 (SE = 0.70).

### 3.3. Role of Education or Socioeconomic Status in Predicting Improvement (Aim 3)

Education and socioeconomic status, general indicators of literacy in Pakistan [70], were assessed as predictors of improvement in PTSD. Years of marriage (a proxy for age, duration of abuse, number of children, etc.) and initial PTSD severity were included as control covariates. Neither education nor socioeconomic status emerged as significant predictors of PTSD improvement (*p* > 0.50 for each), but both shorter marriage duration and lower severity of PTSD symptoms prior to therapy were related to greater reduction in PTSD symptoms (*p* = 0.040 and < 0.001, respectively).

### 3.4. Abuse Characteristics as Predictors of Improvement in PTSD Scores (Aim 4)

In separate regression analyses, we investigated whether (1) severity of various types of abuse and (2) abuser/victim characteristics were related to reduction in PTSD symptoms (Table 3). In each analysis, we included pre-assessment PTSD scores as a control covariate. In the first analysis, the severity of three types of abuse—physical, psychological, and sexual—were considered as predictors of reductions in PTSD. Since preliminary analysis indicated that sexual and psychological abuse were strongly correlated (r = 0.763), these two variables were combined into a single composite variable, “sexual/psychological abuse.” Lower severity of sexual/psychological abuse was significantly associated with greater reduction in PTSD (*p* = 0.047). In contrast, greater severity of physical abuse was associated with greater reduction in PTSD scores (*p* = 0.010).

In a second analysis, intensity of victim/abuser characteristics were entered as predictors of improvement. Neither variable was significant (*p* = 0.609 and 0.697, respectively) (Table 4). Although these two variables were themselves strongly correlated (r = 0.787) (Table 2), neither zero order correlation with PTSD improvement exceeded 0.235.

## 4. Discussion

This analysis provided insight into PTSD experienced by female victims of DA within a cultural tradition that differs substantially from that of the West. Specifically, it revealed that the intensity of PTSD symptoms in such women abated following an imagery-based therapeutic intervention. In addition, the analysis indicated that PTSD reduction was not related to the literacy/education levels of the participants but indicated that the type of abuse may have played a role.

### 4.1. PTSD Following Intervention

The average pre-treatment PCL score for women in this study indicated a fairly high level of burden resulting from DA. Three months after treatment ended, scores had decreased significantly, falling within the typical range for individuals not having PTSD symptomology [71]. Important to this decline was that the amount of improvement was not predicted by the participants’ education or socioeconomic status. Such findings are relevant, as disparity in literacy and resources among subgroups in Pakistani society is substantial, and likely a factor in the effectiveness of treatments that rely primarily on verbal narrative. We believe that therapeutic intervention based heavily on the use of imagery may have been responsible for the general lack of differential effects in participants of different literacy levels.

At the same time, we were surprised by the large effect size evident at the end of the intervention phase. A number of cultural factors and conditions in Pakistan might help explain this outcome. Married women in Pakistan face strong pressure from immediate, in-law, and communal families to conform to traditional roles; they assume heavy domestic responsibilities, including the burden of financial management of a household often under constrained resources. They are expected to bear such burdens without complaint, recognition, sympathy, or assistance, and have few opportunities to interact with supportive friends that might provide cathartic expression and release regarding their experiences [12]. Furthermore, their financial dependence on their husband and extended family may make it impossible to get help without their consent, creating an intractable dilemma for many women. Even if they could independently seek help, the use of psychological services is heavily stigmatized in Pakistan, often associated with family shame and embarrassment. Finally, the mental health system itself is often non-existent, severely under-resourced, or generally unprepared to address the needs of abused women. As a result, women victims of DA represent, for the most part, an invisible/neglected population in Pakistan.

Thus, the eventual realization by these women that the behaviors directed toward them by their husbands were indeed “abusive” and that their strong negative emotional response was, in fact, legitimate, along with the ensuing expression, catharsis, and possible resolution of emotional issues (without financial burden)—so rare an opportunity among abused women in Pakistan—undoubtedly contributed to the strong positive effects of counseling in our sample. Not only did the counseling experience present strategies for dealing with strong emotional fallout, but the supportive therapeutic relationship was likely key to understanding/affirming their feelings, affording them the psychological fortitude to manage the consequences of the DA [72,73]. In this respect, the women’s strong emotional positivity regarding their experience may have led them to overestimate the actual reduction in PTSD symptoms. Interestingly, we found comparable strong positivity (i.e., stronger and more enduring effects) among men in Pakistan with sexual problems who were given the opportunity to supplement pharmacological treatment with “talk-based” therapy, an option rarely afforded Pakistani men experiencing sexual difficulty [74].

### 4.2. Covariates of Improvement

Although our primary objective was to assess the reduction in PTSD from DA following EP intervention, we adopted a “systems” approach to identify contextual covariates that affected the amount of reduction in PTSD symptoms. First, women whose PTSD was lower prior to intervention showed the greatest improvement, a finding that was neither unexpected nor inconsistent with other studies using image-based therapy. Generally, the closer the woman is to the goal at the outset of therapy, the greater the likely benefit [75]. In addition, women who were married for fewer years showed greater improvement, perhaps related to their more limited and less ingrained experience of spousal abuse. In contrast, women who had been in longer relationships may have come to expect patterns of abuse, being more resigned to their lot and showing signs of learned helplessness as characterized by the “battered woman syndrome” [76].

Interestingly, neither characteristics of the abuser nor the victim (as assessed by the KDVSS) predicted the amount of improvement in PTSD symptomology, suggesting that benefits were relatively independent of such factors. However, the type of abuse was related to improvement in PTSD, with those women suffering from lower sexual/psychological abuse showing greater improvement, an unsurprising pattern. In contrast, those women experiencing greater physical abuse also reported greater strides following the intervention. This paradoxical finding may attest to the power of psychological/mental strategies in overcoming the pain/injury related to physical abuse, much like the Buddhist approach of mitigating pain by reframing it as suffering (articulated in the Four Noble Truths; see, for example, https://www.goodnet.org/articles/how-to-cope-suffering-according-buddha (accessed on 2 March 2021), an approach also found in other religious traditions. However, independent verification and interpretation of this pattern is needed.

### 4.3. Coping Strategies and PTSD in Women Victims of DA in Pakistan

Until community interventions are funded, implemented on a national basis, and begin to show positive and enduring effects in reducing the problem of widespread DA in Pakistan, women will continue to suffer privately and in silence, with few options available to them for resolving their feelings and disrupted functioning. For this reason, intervention at the individual level can play an important role in helping victims of DA, both in Pakistan and in similar situations elsewhere. The strides made by women experiencing DA in our sample were particularly notable in view of the fact that, unlike many situations involving spousal abuse, these women were not in a position to leave their relationship, and hence the women risked exposure to continued abuse. Therefore, how is it that women learned to cope with such stressful situations, as indicated by the reduction in PTSD?

Coping typically involves active cognitive/behavioral attempts to either manage the stressful situation (problem-focused) or resolve/reduce the stress (emotion-focused), or both [77]. Problem-focused strategies identify factors responsible for a stressful situation and formulate ways to deal with them (including removing them). Thus, for women experiencing DA, a common solution involves the woman removing herself from the abusive environment, typically leading to separation or divorce. However, for Pakistani women—where cultural traditions differ from Western practices—marital dissolution is usually not a viable option.

In contrast, emotion-focused coping represents a stress-management strategy in which a person focuses on regulating the negative emotional reactions to a stressful situation—rather than attempting to change the stressor itself, the person tries to control her feelings (anxiety) by using a variety of strategies. These strategies may be either positive (e.g., reframing, relaxation, seeking support) or negative (e.g., denial, self-blaming, resignation) and may be the only option available when the stressful situation lies beyond the individual’s control, the case for many Pakistani women in abusive relationships [78]. Indeed, in our sample, the combination of strong social support from the therapeutic relationship/process as well as the therapeutic content itself (which promotes self-esteem, avoidance of self-blame, and emotional regulation) likely represents one of the few viable options currently open to women in abusive relationships in Pakistan. In this regard, we surmise that the image-based therapy supplemented by verbal narratives *and* the support offered through the therapeutic relationship helped in the reduction in symptoms and aided women in re-imagining their role in (and blame for) the abusive actions of their spouse [73], thus mitigating to some extent the consequences of the traumatic events. At the same time, we note that patients’ ongoing dialogue with the therapist during and after treatment suggested that the more composed and calm responses of some women to situations that previously had instigated abuse appeared to mitigate both the likelihood and severity of spousal abuse. This putative process, however, represented only the therapist’s impressions, as formal data regarding ongoing abuse were not collected.

Indeed, a major challenge in this study was the need to circumvent issues of ongoing abuse, that is, therapist–client discussions related to incidents of (ongoing) abuse were generally avoided, given that unpredictable consequences might have resulted had spouses or family members become privy to such dialogue. Hence therapy focused strongly on coping with emotional fallout through treatment alliance, skill building, emotional catharsis and regulation, self-care, and education, largely consistent with the first stage of treatment for complex trauma as described in the literature [79]. Although therapy for PTSD is sometimes not recommended for individuals who continue to be exposed to complex trauma, the question remains at what point should traumatized individuals who are unable to remove themselves from the trauma deserve or be denied treatment. In fact, mental health remediation has a long history of attempting to better the lot of individuals who experience continued exposure to stressors, with stress-management coping strategies often applied in situations where the stressors themselves cannot be removed [77].

Given the above, however, we recognize the critical need for long-term follow-up to ascertain whether treatment benefits were sustained over a longer time interval and/or whether women’s altered responses to their situations truly attenuated the abusive actions/reactions of their spouses, critically important to women unable to remove themselves from an abusive environment due to cultural constraints.

### 4.4. Limitations and Strengths

In lacking a waitlisted comparison group, this pre-post study was limited in the extent to which the reduction in symptomology could be attributed specifically to the EP. In fact, as already noted in the Discussion, we hypothesized that a number of factors, including catharsis, social desirability, acknowledgement of and attention to the participant’s needs/issues, and the patient–therapist alliance probably contributed the reduction in PTSD symptoms. However, the fact that such processes occurred within the context of an intervention supports the idea, that no matter the specific components of the therapy, “talk therapy” for women with PTSD from DA may provide an important source for self-reliance, efficacy, esteem, and at least partial healing [63,72], The fact that women who, on average, had suffered DA for years showed incremental declines in symptoms over the course of 12–15 week, as well as an overall large effect size by the end of the intervention period, strongly argues that the decline in PTSD was not merely the result of the passage of time.

Although a limited sample size (and thus limited power) restricted the number of covariates that could simultaneously be included in the regression analyses, we note that a sample size of 40 women is considerable for an intervention study; indeed, our sample size exceeded that of other studies on women suffering complex trauma from DA who participated in a therapeutic intervention (e.g., [80]). Furthermore, the generalizability of our findings to all women with PTSD from DA in Pakistan is limited by the sample characteristics, although on the positive side, our sample did show ranges of age, socioeconomic class, and education. The study would have benefitted from inclusion of an untreated or waitlisted control group, post-test assessments implemented by a neutral third party so as to avoid social desirability bias, and the addition of relevant covariates, such as select co-morbidities, frequency of ongoing abuse, relationship quality, and other environmental social stressors.

## 5. Conclusions

Women of varying levels of economic class and literacy in Pakistan suffering PTSD from DA showed significant decreases in symptomology following intervention using an image-based therapy. Women’s initial (pre-treatment) level of symptomology, years of marriage, and type of abuse predicted amount of improvement. The importance of understanding and addressing cultural issues related to abuse and treatment are critical to achieving positive outcomes.

## Figures and Tables

**Table 1:** Frequencies (f) and percentages (%), or mean and standard deviation for demographic characteristics of participants (N = 40).

Variable	Mean ± SD or Categories	f	%
Age	34.3 (7.47)		
Education	Secondary	9	22
	Intermediate	7	17
	Graduate	11	27
	Postgraduate	13	32
Occupation	Housewife	25	62
	Employed	15	37
Monthly Income	20,000–30,000 (USD 119–179)	3	7
	31,000–50,000 (USD 185–298)	9	22
	51,000–60,000 (USD 304–358)	10	25
	61,000 < (USD 364–above)	18	45
Family System	Nuclear	16	40
	Extended	24	60
No. of Children	1.8 (1.22)		
Years of Marriage	8.8 (6.49)		
Residential Area	Lower class	5	12
	Middle class	15	37
	Upper middle class	11	27
	Upper class	9	22

**Table 2:** Spearman correlations among predictor variables and PCL change score.

Variables	Age	Education	Years Married	Residential Area	Physical Abuse	Sexual Abuse	Psychological Abuse
PCL Change	0.068	0.237	−0.259	0.297	−0.254	0.028	0.154
Age		0.225	0.531 ***	0.161	0.073	0.153	0.127
Education			0.179	0.455 **	0.076	0.336 *	0.293
Years Married				−0.024	0.054	0.064	0.111
Residential Area					−0.037	0.022	−0.048
Physical Abuse						0.668 ***	0.525 ***
Sexual Abuse							0.763 ***

Note: * *p* < 0.05, ** *p* < 0.01, *** *p* < 0.001.

**Table 3:** Regression results for predicting PCL improvement (change) based on type of abuse.

	PCL Improvement (Change)	
Predictor	Coefficient (SE)	*p*-Value
PCL Pre-Assessment Score	−1.030 (0.026)	0.001
Physical Abuse	0.278 (0.130)	0.010
Sexual/Psychological Abuse	−0.059 (0.029)	0.047

Note: Significant *p*-values (*p* < 0.05) are in bold. Adjusted R^2^ = 0.138 (*p* = 0.024).

**Table 4:** Predictors of PCL improvement (change) based on victim and abuser characteristics.

	PCL Improvement (Change)	
Predictor	Coefficient (SE)	*p*-Value
PCL Pre-Assessment Score	−1.013 (0.027)	0.001
Abuser Characteristics	0.081 (0.157)	0.609
Victim Characteristics	−0.065 (0.166)	0.697

Note: Significant *p*-values (*p* < 0.05) are in bold. Adjusted R^2^ = 0.023 (*p* = 0.573).

## Data Availability

Data can be made available upon reasonable request.

## Supplementary Materials

The following are available online at https://www.mdpi.com/1660-4601/18/5/2478/s1, Table S1: Eidetic Trauma Model (ETM) Session Structure, Table S2: Steps 5–17 of the Trauma Model.
