# Supplementary material for: Possible Role for Imagery-Based Therapy in Managing PTSD in Pakistani Women Experiencing Domestic Abuse: A Pilot Study Using Eidetic Therapy
Source: Int J Environ Res Public Health. 2021 Mar 3;18(5):2478. doi: 10.3390/ijerph18052478 (PMC7967602; doi:10.3390/ijerph18052478)
Supplement: Supplementary file 1 [file ijerph-18-02478-s001.pdf]

Supplementary Materials

Table S1. Eidetic Trauma Model (ETM) Session Structure

**Sessions**	**Session Content**	**Phases**
Session 1	Introductory Session<br>Intake History<br>Screening of PTSD-PCL<br>Screening of DV- KDVSS	Pre-Intervention Phase
Session 2–3	Orient the Client to Eidetic Therapy (ETM)<br>Set Initial Treatment Plan/Goals<br>Preliminary Information on Trauma	
Session 4–5	Levels of Inquiry<br>Application of Trauma Model Treatment<br>Begin Intervention—Accident Trauma (Step 5–8)	Intervention Phase
Session 6–7	Continue Intervention (Step 9–11)	
Session 8–9	Continue Intervention (Step 12–15)	
Session 10–11	Continue Intervention (Step 15–17)	
Session 12–13	Check Symptoms and continue or discontinue intervention	
Session 14–15	Follow up – after 3 months<br>Applied PCL to reassess symptoms	Post Intervention Phase
Session 15–16	Discuss ending treatment—closing Session	

Table S2. Steps 5–17 of the Trauma Model

**S#**	**Event Trauma (Steps 5–17)**
5	Event Trauma Imagery
6	Pre-Diurnal Event Images
7	Pre-Near Event Images
8	Pre-Synchronous Event Images
9	Mid Event Impact Images
10	Mid Event Control Images
11	Mid Event Loss of Control Images
12	Mid Event Death Images
13	Mid Event Support Images
14	Mid Event Exertion Images
15	Post Near Event Images
16	Post Diurnal Event Images
17	Post Remote Event Images
